# Endoscopic endonasal approach for MRI–Negative Cushing’s microadenoma

**DOI:** 10.3171/2023.4.FOCVID2324

**Published:** 2023-07-01

**Authors:** Jeffrey J. Feng, Stephanie K. Cheok, Alexander G. Chartrain, John D. Carmichael, Mark S. Shiroishi, William J. Mack, Gabriel Zada

**Affiliations:** 1 Department of Neurosurgery, Keck School of Medicine of University of Southern California, Los Angeles;; 2 University of Southern California Brain Tumor Center, Los Angeles;; 3 University of Southern California Pituitary Center, Los Angeles, California; and; 4 Western Michigan University Homer Stryker M.D. School of Medicine, Kalamazoo, Michigan

**Keywords:** pituitary adenoma, cavernous sinus wall, 7T MRI, tumor resection, MRI–Negative Cushing’s disease

## Abstract

A 54-year-old male with a history of diabetes mellitus type 2 for 12 years and hypertension was seen in the clinic due to poorly controlled diabetes. Inferior petrosal sinus sampling (IPSS) confirmed Cushing’s disease with primary adrenocorticotropic hormone (ACTH)–secreting pituitary adenoma on the right. However, 3T and subsequent 7T MRI showed no visible tumor. An endoscopic transsphenoidal approach was selected to explore the pituitary gland and resect the presumed microadenoma. Tumor was identified in the lateral recess along the right medial cavernous sinus wall and gross-total resection (GTR) was performed. The normal pituitary gland was preserved, and the patient went into remission.

The video can be found here: https://stream.cadmore.media/r10.3171/2023.4.FOCVID2324

## Transcript

This surgical video demonstrates the techniques for a safe endoscopic transsphenoidal resection of a pituitary microadenoma in a patient with MRI–Negative Cushing’s disease.

### 0:30 Background.

The surgical approach differs from conventional resection of pituitary adenomas visible on MRI, as emphasis is placed on the exploration of the pituitary gland and the cavernous sinus while preserving as much of the normal gland as possible.^1^

### 0:42 Patient Presentation.

The patient is a 54-year-old male with a history of type 2 diabetes for 12 years and hypertension presenting to the clinic with poorly controlled diabetes. Additional cushingoid features including increased abdominal girth, facial plethora, and severe limb girdle weakness prompted evaluation for Cushing’s disease. Plasma, 24-hour urine free, and late-night cortisol levels^2^ were elevated, and initial 3T MRI and subsequent 7T MRI^3–5^ were negative. CT of the abdomen also showed a small adrenal mass. Inferior petrosal sinus sampling (IPSS) confirmed the presence of a primary ACTH-secreting adenoma on the right side of the pituitary gland. Endoscopic transsphenoidal surgery for pituitary gland exploration and adenoma resection was indicated.^6^

### 1:25 Preoperative MRI.

Shown here are the 7T MRI images of the patient’s brain, which were taken after the standard 3T MRI was negative. This was also negative as only normal pituitary gland, stalk, and the optic chiasm are visible.

1:35 Diagnostic Role of IPSS. A crucial piece of our diagnostic workup included an inferior petrosal sinus/cavernous sinus sampling test. IPSS is indicated when initial diagnostic MRI is equivocal or negative.^1,7^ Venous blood is sampled at the right and left cavernous sinus along with the peripheral femoral vein before and after intravenous injection of desmopressin to stimulate ACTH production. This is done by introducing a catheter through the common femoral vein and into the jugular vein. A microcatheter is then navigated through the inferior petrosal and into the cavernous sinus on each side. Significant elevation and ACTH levels from the blood sample on one side, as was present in this case on the right (compared to left and peripheral), confirms the pituitary source of hypercortisolism and the laterality. The ACTH values further increased following desmopressin injection. With this information we know that surgical exploration should focus on the right side of the pituitary gland.

### 2:26 Patient Positioning.

The patient was identified by staff and induced and intubated by anesthesia. He was then placed in a supine position with the head and back elevated at 30°, secured by a 3-pin fixation device, and the head tilted toward the surgeon at 15°. The nasal cavity was prepped with oxymetazoline and povidone iodine, and the abdomen was prepped for potential fat graft.

### 2:45 Initial Exposure at the Sellar Floor.

Following initial exposure of the nasal cavity, our video begins with the endoscope in the sphenoid sinus directly facing the sella. The sellar floor has been partially removed with a Kerrison rongeur, extending to the cavernous sinuses. Notice that a greater portion of the right parasellar floor has been exposed as a prior IPSS evaluation determined that the source of primary ACTH secretion was on the right side.^5^

### 3:09 Locating the Internal Carotid Arteries.

A neuronavigation probe is then introduced to identify the midline of the sella and the relative position of the internal carotid arteries. A micro-Doppler probe is then used to map out the location of the internal carotid arteries bilaterally, especially the right carotid artery.

### 3:23 Dural Incision and Entry Into Sella.

An 11 blade was then used to make a cruciate incision in the sellar dura. Intercavernous sinus bleeding was expected and managed with hemostatic agents and light pressure. A nerve hook was used to open the dura further, and an arachnoid knife was used to extend the incision over the right carotid artery and the right cavernous sinus.

### 3:40 Exploration of the Pituitary Gland, Medial Cavernous Sinus Wall, and Resection of Tumor.

The attachments of the pituitary gland were separated from the medial cavernous sinus wall. We then explored the right inferior aspect of the pituitary gland. With a small ring curette, we were able to identify fragments of white-appearing adenoma approximately 3 mm in size (highlighted in green). We used endoscopic scissors to resect the medial cavernous sinus wall^8^ to open the recess through which we used our ring curette to identify the boundaries of the tumor (highlighted in green) and remove it. Prior to closure, we explored this area and determined that we were able to achieve gross-total resection.

### 4:13 Contingency Plan if No Tumor Were Found.

Had we not found tumor on this side, our plan was to continue our exploration of the left side of the pituitary gland in similar fashion. If we were still unable to find any tumor tissue, we would proceed with hemi-resection of the pituitary gland on the right side.

### 4:25 Skull Base Repair and Surgical Closure.

Much of the normal pituitary gland (highlighted in blue) was left in place. A Valsalva maneuver was performed, and there was no evidence of CSF leak. We then proceeded with repair and closure of the surgical cavity with a monolayer of hemostatic cellulose and sponges soaked in thrombin to fill the dead space. The sponges were held in place with another layer of hemostatic cellulose in cargo net fashion. Some bioadhesive was used. There was no evidence of bleeding or CSF leakage. Meticulous hemostasis was then achieved in the sphenoid sinus and nasal cavity. The choanae were suctioned out, and the turbinates were medialized.

### 5:00 Postoperative Course.

The patient woke up from anesthesia without neurological deficits and had a stable postoperative course. He was discharged on the 3rd day after surgery. Postoperative cortisol trended downward^2,5^ from 4.1 μg/dL on the evening following surgery down to 2.6 the next morning on postoperative day 1. The nadir level of 2.6 μg/dL was consistent with early remission. Surgical pathology confirmed ACTH adenoma. Aside from the steroid supplementation, which we planned to taper over months, no other hormone medications were necessary, and this was consistent with our surgical approach to preserve the normal pituitary gland. He continued insulin but was able to wean off all his previous antihypertensive medications.

The patient reported that his weakness and fatigue have improved on the first clinical visit, and that healing was progressing well without CSF leak. He continues to do well and regain strength at his recent 3-month follow-up visit.

### 5:49 Postoperative MRI.

His 3-month postoperative MRI, displayed on the right, shows an intact pituitary gland and stalk with no evidence of residual.

This concludes our video. Thank you.

## Acknowledgments

We wish to thank Rebekah Ghazaryan, our outpatient nurse at the University of Southern California Brain Tumor Center, for her assistance in retrieving the patient’s 7T imaging.

## Disclosures

The authors report no conflict of interest concerning the materials or methods used in this study or the findings specified in this publication.

## Author Contributions

Primary surgeon: Zada. Assistant surgeon: Cheok, Chartrain, Mack. Editing and drafting the video and abstract: Zada, Feng, Chartrain. Critically revising the work: all authors. Reviewed submitted version of the work: Zada, Feng, Cheok, Carmichael, Shiroishi. Approved the final version of the work on behalf of all authors: Zada. Supervision: Zada, Chartrain, Carmichael.

## Supplemental Information

### Patient Informed Consent

The necessary patient informed consent was obtained in this study.
